# 3D-Arterial analysis software and CEUS in the assessment of severity and vulnerability of carotid atherosclerotic plaque: a comparison with CTA and histopathology

**DOI:** 10.1007/s11547-022-01551-z

**Published:** 2022-09-17

**Authors:** Daniele Fresilli, Nicola Di Leo, Ombretta Martinelli, Luca Di Marzo, Patrizia Pacini, Vincenzo Dolcetti, Giovanni Del Gaudio, Fabrizio Canni, Ludovica Isabella Ricci, Corrado De Vito, Corrado Caiazzo, Raffaella Carletti, Cira Di Gioia, Iacopo Carbone, Steven B. Feinstein, Carlo Catalano, Vito Cantisani

**Affiliations:** 1 Department of Radiological, Oncological, and Pathological Sciences, Sapienza University of Rome, Viale Regina Elena 324, 00161 Rome, Italy; 2 Department of Surgery “Paride Stefanini’’, Vascular and Endovascular Surgery Division, Sapienza University of Rome, Viale del Policlinico 155, 00161 Rome, Italy; 3 Department of Public Health and Infectious Diseases, Sapienza University of Rome, 00185 Rome, Italy; 4 Breast Service, Local Health Agency of Naples ASL NA1, Naples, Italy; 5 Department of Translational and Precision Medicine, Sapienza University of Rome, Viale Regina Elena 324, 00161 Rome, Italy; 6 Department of Radiological, Oncological and Pathological Sciences, Diagnostic Imaging Unit, ICOT Hospital, Sapienza University of Rome, Via Franco Faggiana1668, 04100 Latina, Italy; 7 Department of Internal Medicine, Section of Cardiology, Rush University Medical Center, Chicago, IL USA

**Keywords:** Carotid plaque; carotid stenosis, CEUS, Neovascularization, Atherosclerosis

## Abstract

**Purpose:**

Our purpose is to assess Multiparametric Ultrasound (MPUS) efficacy for evaluation of carotid plaque vulnerability and carotid stenosis degree in comparison with Computed Tomography angiography (CTA) and histology.

**Material and methods:**

3D-Arterial Analysis is a 3D ultrasound software that automatically provides the degree of carotid stenosis and a colorimetric map of carotid plaque vulnerability.

We enrolled 106 patients who were candidates for carotid endarterectomy. Prior to undergoing surgery, all carotid artery plaques were evaluated with Color-Doppler-US (CDUS), Contrast-Enhanced Ultrasound (CEUS), and 3D Arterial analysis (3DAA) US along with Computerized Tomographic Angiography (CTA) to assess the carotid artery stenosis degree. Post-surgery, the carotid specimens were fixed with 10% neutral buffered formalin solution, embedded in paraffin and used for light microscopic examination to assess plaque vulnerability morphological features.

**Results:**

The results of the CTA examinations revealed 91 patients with severe carotid stenoses with a resultant diagnostic accuracy of 82.3% for CDUS, 94.5% for CEUS, 98.4% for 3DAA, respectively. The histopathological examination showed 71 vulnerable plaques with diagnostic accuracy values of 85.8% for CDUS, 93.4% for CEUS, 90.3% for 3DAA, 92% for CTA, respectively.

**Conclusions:**

The combination of CEUS and 3D Arterial Analysis may provide a powerful new clinical tool to identify and stratify “at-risk” patients with atherosclerotic carotid artery disease, identifying vulnerable plaques. These applications may also help in the postoperative assessment of treatment options to manage cardiovascular risks.

## Introduction

Cerebral vascular accidents (CVA) are estimated to be the second cause of death in Europe, and globally resulting in 405,000 deaths (9%) in men and 583,000 (13%) deaths in women each year [1]. Thus, CVA remains a massive public health problem and requires better strategies for the prevention and treatment of this disease.

Approximately 15% of all first-ever CVA occur due to atheroembolism from an asymptomatic carotid stenosis which can potentially be detected by effective imaging techniques and prevented by carotid artery revascularization [2].

The vulnerable plaque is defined as a plaque prone to rupture and susceptible to vascular complications as arterial occlusion and/or distal embolism. One of the first authors to use the term “vulnerable plaque” was Muller about coronary plaque [3], and these concepts have been translated into extracranial carotid arteries in subsequent scientific literature studies [4]. A complete list of criteria was proposed for defining vulnerable plaques with major criteria (inflammatory cells infiltration, thin cap with large lipid core, endothelial fissuration, aggregated platelets on superficial plaque surface, high stenosis grade) and minor criteria (superficial calcified nodule, intraplaque hemorrhage, endothelial dysfunction, outward positive remodeling) [5].

The standard of care based on professional society guidelines recommends carotid artery revascularization treatment for the management of patients with symptomatic carotid artery stenosis > 50% [2]. For asymptomatic patients, the revascularization is considered if the luminal stenosis > 60% and the patients are considered at high long-term risk for a CVA [2]. Importantly, over the last decade, the best medical therapy (BMT) reduced the annual risk of CVA in patients with asymptomatic carotid artery stenosis to approximately 0.5% with the comparative advantages of carotid artery revascularization less clear. The inherent surgical risks, either endarterectomy (CEA) or stenting (CAS), require particular clinical consideration in patients with underlying increased CV risks and associated high-risk atherosclerotic carotid artery plaques[6]. Previously, the criteria for surgical revascularization or stent placement were based primarily on the presence and degree of arterial lumen narrowing as the sole parameter. This anatomic parameter may not be adequate today based on a need to incorporate the tissue characteristics of the plaque that includes plaque composition and morphology [7–9]. Based on the underlying dynamic physiology of a vulnerable plaque, as opposed to reliance only on anatomic descriptors, the plaque may spontaneously rupture resulting in a catastrophic CV event [10] resulting in a catastrophic event despite the defined presence of a low-grade stenosis. Histologically, the vulnerable carotid plaque is characterized by increased presence of inflammatory infiltrates, lipid-rich necrotic cores (LRNC), intraplaque neovascularization (IPN) and thin fibrous cap [11]. The disruption of newly formed vasa vasorum within the plague may result in spontaneous intraplaque hemorrhage (IPH) and thrombosis [11]. Additionally, the presence of a thin cap with microcalcification increases plaque vulnerability, weakening the plaque and contributing to rupture and ulceration [12].

Therefore, the paradigm shift from sole reliance on vessel anatomy (degree of carotid stenosis) now includes physiologic markers of plaque vulnerability parameters that more accurately assess the overall cardiovascular risk of patients, including CVA risk.

Color-Doppler ultrasound (CDUS) imaging is the most widely used imaging modality to detect the presence and severity of carotid plaques. Although in recent years, several ultrasonographic techniques have been developed and include contrast-enhanced ultrasound (CEUS) [13–18], elastosonography [19, 20] and 3D Ultrasound [21], collectively termed Multiparametric Ultrasound (MPUS) [22]. The combination of parameters has emerged as valuable imaging tool for assessing plaque morphology and composition and refines the assessment of anatomic stenosis.

Specifically, the implementation of ultrasound contrast ultrasound (CEUS) administration improves conventional techniques particularly in cases associated with slow flow luminal flow in patients with a critical carotid stenosis.

CEUS has been shown to enhance and exceed the performance of CDUS for the determination of carotid sub-occlusive stenoses distinguishing from a total carotid occlusion, thus resulting in improved evaluation of the internal vascular surface and carotid ulcerations [23, 24].

CEUS has been shown to detect plaque neovascularization [25] within newly formed plaques or plaques that reveal significant inflammation [13, 26].

The novel applications of 3D US examination utilize 3D software resulting in volume representation of plaque conformation, shape, and structure, while providing a 3D model in three spatial planes. The procedure utilizes conventional 2DUS with a multifrequency 3–14 MHz probe that is briefly held in position over the plaque of interest. The software provides a read out of quantitative analysis of maximum stenosis and plaque volumes.

Computed tomography angiography (CTA) is widely accepted and used as for carotid stenosis measurements. The relatively high resolution permits an assessment of plaque ulcerations [27]. The advantages of using CTA include availability, restively rapid data acquisition and a Z-axis parameter that provides enhanced spatial resolution and three-dimensional reconstruction [27]. Known limitations of CTA include the beam-hardening artifact that results in plaque stenosis overestimation, the use of iodinated contrast agents and the radiation exposure.

Differentiating advantages of using ultrasound include low cost, an established safety profile, wide end user applications and real-time assessment of pathology. We hypothesis that implementation of the newer 3D volumetric software (MPUS) will result in improved management of patients with carotid artery disease and consequently avoid redundancies of using CTA. Of note, the safety profile of the CEUS is enhanced as compared to the use of iodized agents (CTA) and gadolinium-based exposures used in MRA [28, 29]. Although, the clinical applications of MPUS have not be fully appreciated nor represented in the most recent European Society for Vascular Surgery (ESVS) 2023 guidelines [30].

In order to support our contention that MPUS is a valuable clinical modality with distinct and unique applications, we choose to perform a direct comparison of the diagnostic accuracy of MPUS with Color-Doppler ultrasound (CDUS), CEUS and 3D ultrasound using 3D Arterial Analysis software (3DAA) as compared to (CTA) for carotid stenosis. Our assessment includes an analysis of plaque histology as a determinant of carotid plaque vulnerability.

## Materials and methods

106 consecutive patients scheduled to undergo carotid endarterectomy between June 2016 and December 2021 at the Department of Surgery “Pietro Valdoni,” Sapienza University of Rome (Italy) were included in the study. The study was approved by the Ethical Committee of “Sapienza” University of Rome, in accordance with the declaration of Helsinki and the Guideline for Good Clinical Practice. Before initiating the study, all participants provided a written informed consent for the intervention.

Before surgery, all patients underwent a pre-operative evaluation with MPUS (CDUS, CEUS, 3D Arterial Analysis) and CTA. CTA was performed within two days and two weeks following MPUS evaluation. Inclusion criteria were defined as a carotid stenosis ≥ 50% in symptomatic patients or carotid stenosis ≥ 60% in asymptomatic patients. Five patients were excluded from the study due to the presence of coarse carotid calcification (1 patient), short neck conformation (1 patient), increased hypoechoic plaques not recognized by 3D software (2 patients) and in one patient, the CTA examination was performed without iodinate contrast medium due to patient contraindications (1 patient).

Overall, carotid ultrasound evaluations were performed in 101 patients using high-end equipment (SAMSUNG, RS80 and 85 Prestige). All examinations were performed by radiologist with extensive experience with 15 years of experience using CEUS (V.C.). The examination was performed with two different probes and included a linear probe (model L3-L12) with a frequency of 7.5 MHz, for the Color-Doppler US and CEUS evaluation, and a volumetric linear probe (model LV3-14; mechanical type) with a frequency between 3 and 14 MHz, for 3D US evaluation. The examinations were reviewed independently by another radiologist, blinded to clinical information and US reports, performing and/or reviewing CTA imaging (D.F.).

### Diagnostic modality

#### US and CDUS

**Fig. 1:**
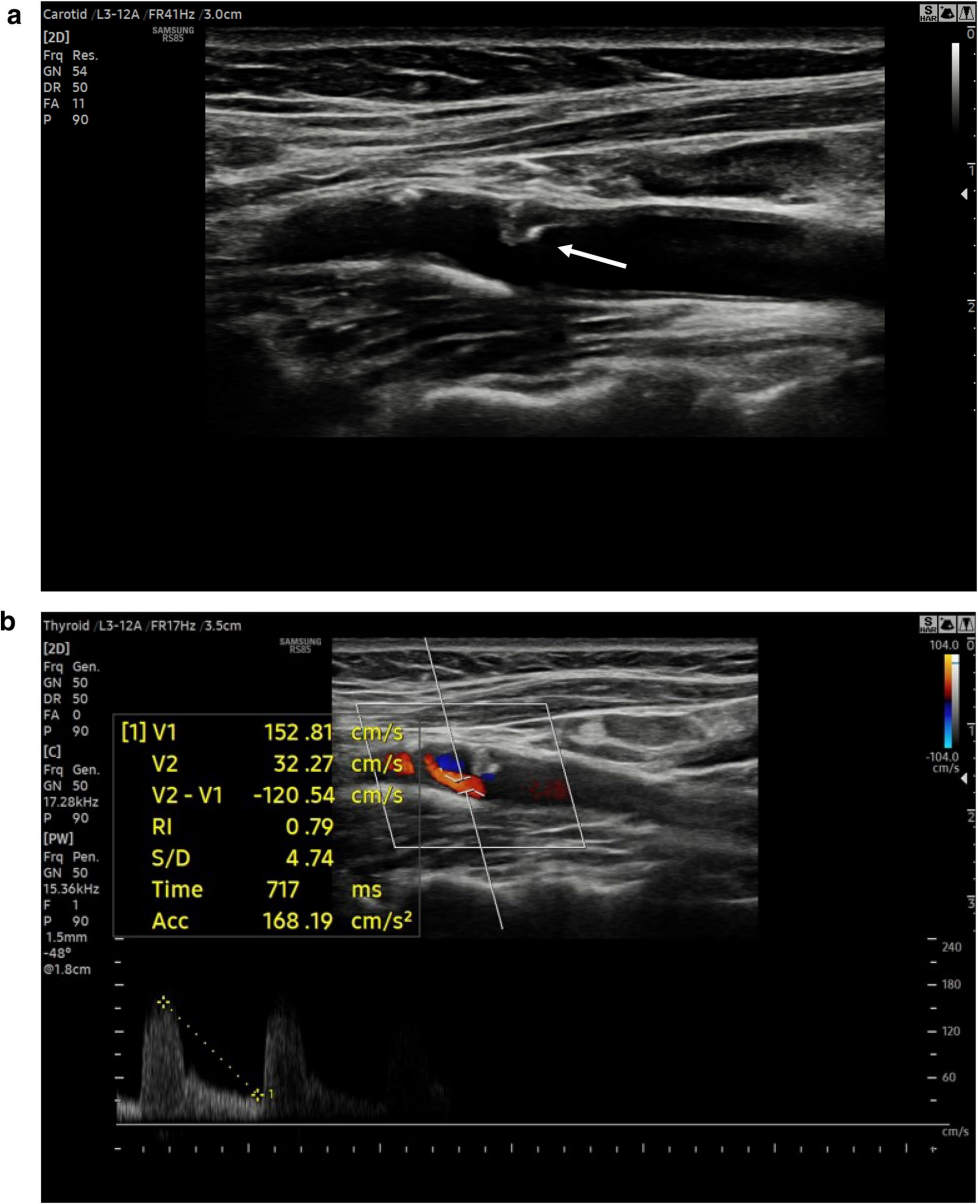
**a** At baseline US, an inhomogeneous non-calcified plaque (arrow) is detected at left internal carotid artery with a stenosis degree between 50–60% in asymptomatic patient. **b** Doppler spectrum shows a mild elevated systolic velocity peak (153 cm/s) confirming a carotid stenosis degree between 50 and 70%

Patients were imaged in a supine position with a pillow under their shoulders to allow neck hyperextension and turned toward the contralateral side. Axial and longitudinal sonograms were acquired to estimate the grade of stenosis and to characterize the morphology of the plaque. The study population was divided into the following categories: < 50% stenosis, 50–69% stenosis, and ≥ 70% stenosis. The *carotid plaque* was considered *severe* if associated with ≥ 70% lumen decrease and *vulnerable* if showed > 50% hypoechoic areas (Gray-Weale type I or II [31]) and/or superficial ulceration (Fig. **1****a-b**).

#### CEUS

**Fig. 2:**
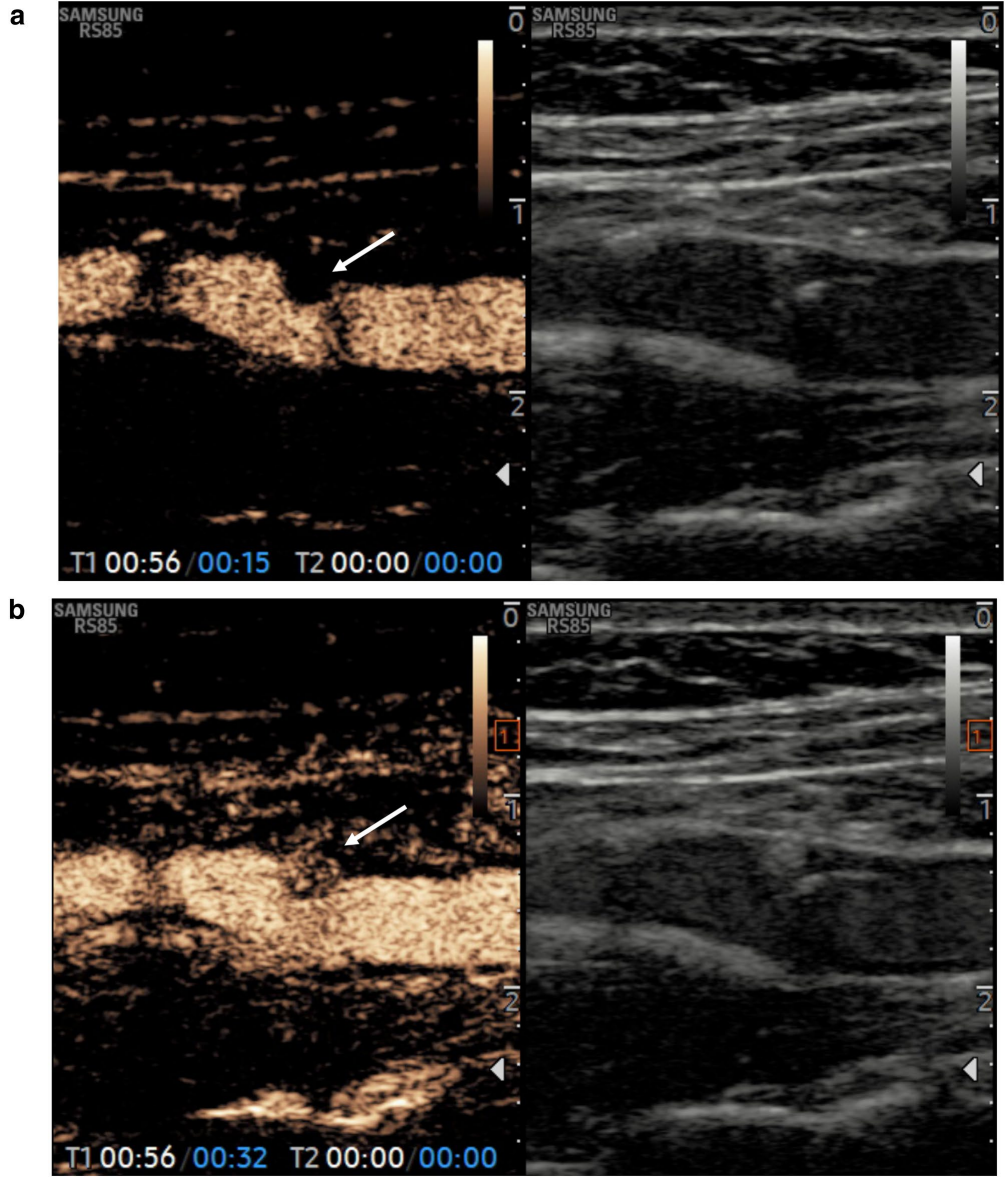
**a** CEUS shows a carotid stenosis degree about 50% at the left internal carotid artery and not eligible for surgery or stenting. Plaque surface is mild irregular (arrow). **b** After few seconds, CEUS shows a marked intraplaque neovascularization (arrow) (grade III) suggesting a vulnerable plaque and eligible for surgery or stenting

CEUS was performed after bolus injection of 1,2 ml of SonoVue (Bracco, Milan, Italy) through a 20-gauge cannula into an antecubital vein, followed by a 10 ml saline flush. The carotid plaque was scanned to permit visualization of the entire plaque volume and surrounding arterial walls. The evaluation continued for at least 2 min, using a non-destructive US mode with low MI (MI 0.06–0.08). The video clip of the procedure was digitally recorded for further analysis. Qualitative analysis was performed assigning a score in a scale from 1 to 3 as following: absence of contrast enhancement (score 1), enhancement confined to the adventitial or peripheral region of the plaque (score 2), diffuse intraplaque contrast enhancement (score 3). The *carotid plaque* was considered *severe* for stenosis degree ≥ 70% and *vulnerable* if presented superficial ulceration and/or adventitial or internal plaque enhancement (score 2–3) (Fig. **2****a-b**).

#### 3D arterial analysis software

**Fig. 3:**
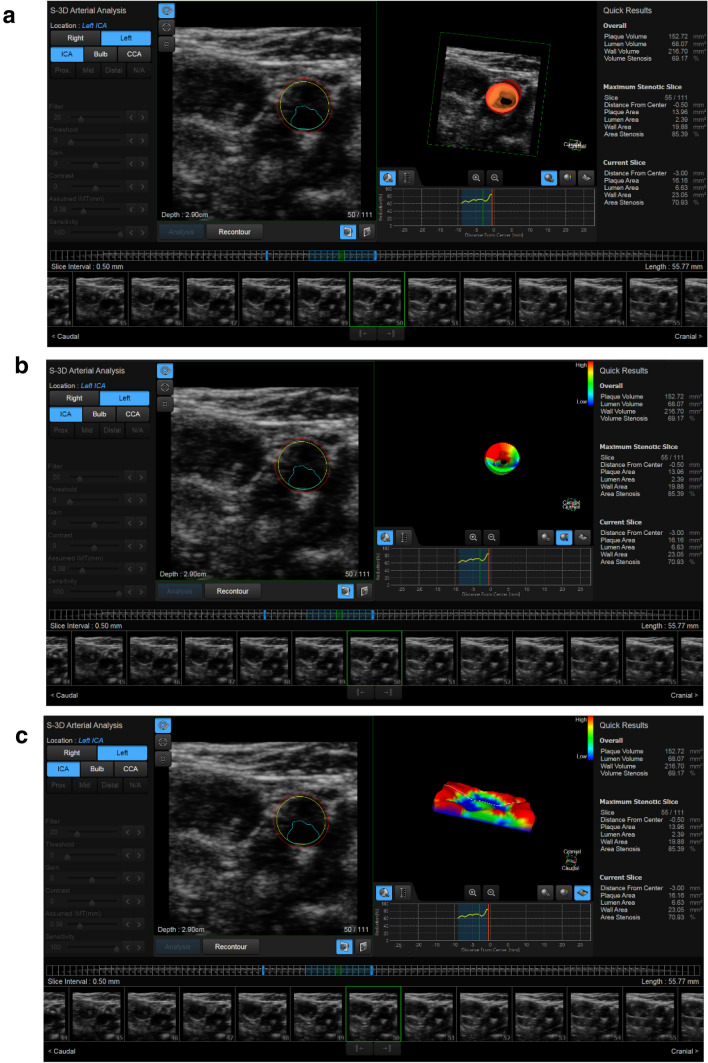
**a** 3D Arterial Analysis shows the mild/severe stenosis (69%) at left internal carotid artery in a volumetric evaluation. **b** 3D Arterial Analysis shows the plaque chromatic map according to the plaque vulnerability in a volumetric evaluation. The prevalence of the red areas indicates a soft and vulnerable plaque. **c** 3D Arterial Analysis shows the same plaque chromatic map in a superficial evaluation

With patient in the same position, a 3D US examination was performed. A multifrequency 3–14 MHz probe was used for the study and the 3D Arterial analysis software provided both quantitative analysis and 3D reconstruction. For these studies, the operator placed the probe at the plaque level, maintaining the position for about 3 s and avoiding to apply pressure on the neck to minimize the compression artifacts. The applied software highlighted the area of interest and allowed the operator to define the stenosis degree and the blood flow reduction. Moreover, the software was able to show plaque conformation, shape and structure, providing a 3D model evaluable in each one of the three spatial planes. The application of the software also provided a plaque colorimetric map relative to major or minor vulnerability areas (red areas were softer with a higher content of lipids). Based on the 3D arterial analysis, *vulnerability criteria* were decided as a major presence (> 50%) of red areas than blue areas (Fig. **3****a-c**).

#### CTA

**Fig. 4:**
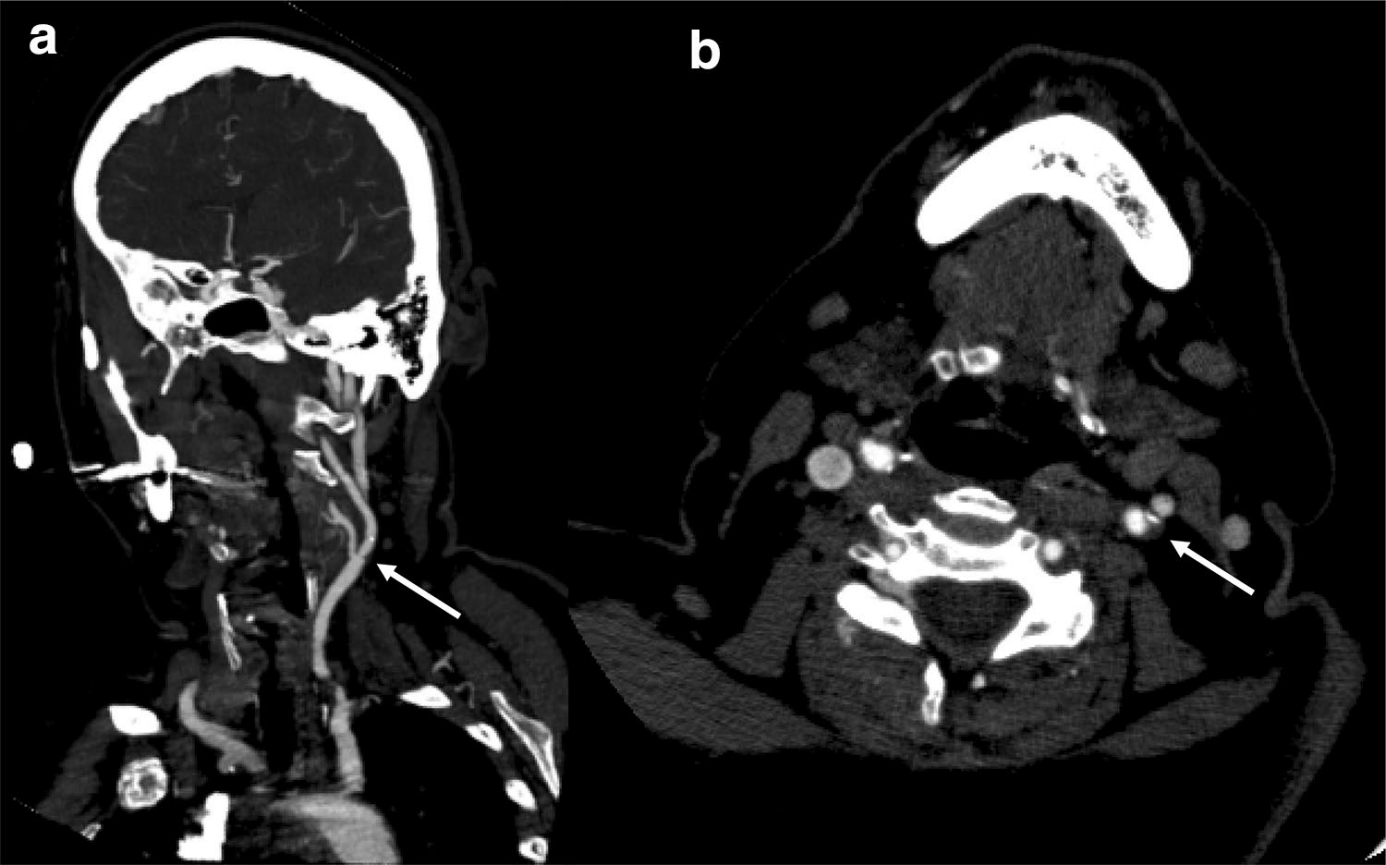
At CTA, the left carotid plaque seen on the previous ultrasound examination (arrows) is predominantly fibrotic and determines a mild/moderate carotid stenosis (40–50%); **a** coronal plane. **b** axial plane. Intraplaque neovascularization is not present

CTA was performed using a Sensation 64 (Siemens, Erlangen, Germany) or Ingenuity 128 (Philips, Amsterdam, Netherlands). A two-phased CT protocol was used, with a pre-contrast phase and an arterial phase (initiated with bolus tracking). 80 mL of non-ionic contrast agent (Iopamiro 370 mg/ml, Bracco, Milan, Italy) was injected at 3.5–4 mL/s with a double-head injector through an 18- to 20-gauge IV cannula in an antecubital vein, followed by a 50 mL saline bolus. The other scanning parameters were: 1.2 mm acquisition; reconstruction with a soft-margin kernel algorithm (B20) at 1.5 and 3 mm; pre-contrast scans at a low-power tube (120 mAs); the arterial phase at 120 kVp and 200 mAs. Coronal and oblique reconstructions along the longitudinal axis of carotids were obtained and the stenoses were calculated with NASCET method (Fig. **4**). *Vulnerable plaques* were considered those with at least one of the following criteria: absence of calcifications accounting for > 50% of the plaque, negative HU values at pre-contrast CT-scan and > 20 HU enhancement in post-contrast CT-scan.

#### Histological and immunohistochemical evaluation

After surgery, the carotid endarterectomy specimens were immediately fixed with 10% neutral buffered formalin solution for histological examination. The calcified plaques underwent 24-h decalcification. Paraffin-embedded tissue Sects. (3 μm) stained with hematoxylin–eosin were used for light microscopic examination to assess morphological features of carotid plaque: composition (fibrous vs atherogenic), lipid core (absence/presence); thickness of the fibrous cap (μm), ulceration (absence/presence); calcifications (absence or presence and deep or superficial), intraplaque inflammatory infiltrate, microvessels and hemorrhage (absence/presence).

The lipid core was graduated as absent, minimal, moderate or extensive wideness.

The fibrous cap was considered thin with thickness value < 200 μm and thick if ≥ 200 μm.

The characterization of intraplaque inflammatory infiltrate was performed on consecutive paraffin-embedded tissue sections of each case by immunohistochemistry with using BOND-III automated IHC stainer (Leica Biosystems, Milan, Italy) with ready-to-use antibody (Novocastra, Newcastle upon Tyne, UK), respectively, anti-CD3 for T lymphocytes and anti-CD68 for macrophages, and HRP-DAB detection system; a semiquantitative evaluation of the inflammatory cells (absent, minimal < 5 inflammatory cells, moderate 5–10 inflammatory cells and extensive > 10 inflammatory cells) was performed by two independent pathologists.

For each case, the evaluation of intraplaque micro-vessel density was performed with CD34 immunohistochemical stain, specific marker for endothelial cells, on consecutive paraffin-embedded tissue sections using BOND-III automated IHC stainer (Leica Biosystems, Milan, Italy) with CD34 ready-to-use antibody (Novocastra, Newcastle upon Tyne, UK) and HRP-DAB detection system; all immunostained slides was captured with Aperio scanner (Leica Biosystems, Milan, Italy), and five selected images at 20X magnification for each slide were used to value the intraplaque number of vessels (CD34 +) by a computerized imaging software (Image J, NIH, Bethesda, MD) and expressed as vessel number/total area (micro vessel density).

The carotid plaque was considered morphologically *vulnerable* if it presented a moderate-extensive lipid core, a thin fibrous cap, moderate-extensive inflammatory infiltrate, and high value of intraplaque microvessel density.

### Statistical analysis

The statistical analysis evaluated the accuracy of the different modalities used to identify patterns associated with vulnerability of the atherosclerotic plaque. Sensitivity, specificity, PPV, NPV and area under the curve (AUC) were calculated. Differences between AUCs of different imaging methods were evaluated through Bonferroni test. A p-value < 0.05 was considered statistically significant. Statistical analysis was carried out with Stata software (Stata v. 15, Statacorp LLC, College Station TX, USA).

## Results

### CTA

For purposes of standardization, the CTA was considered the standard used for the carotid artery stenosis, resulting in a total of 91 plaques with high grade of stenosis.

62 out of 71 plaques were considered vulnerable at CTA and all specimens were confirmed at histology. 29 out of the 30 plaques were confirmed as stable, with one false positive case.

These results indicted a sensitivity of 87.3% (95% confidence interval (CI), 77.3–94.0%), with a specificity of 96.7% (95%CI, 96.7–99.9%) and a PPV of 98.4% (95%CI, 91.5–100%) and a NPV of 76.3% (95%CI, 59.8–88.6%). AUC was 92% (95%CI, 87–97%).

### CDUS

77 out of the 91 plaques with high grade of stenosis (CTA confirmed) were demonstrated at CDUS, with a sensitivity of 84.6% (95%CI, 75.5–91.3%), a specificity of 80.0% (95%CI, 44.4–97.5%), a PPV of 97.5% (95%CI, 91.2–99.7%), a NPV of 36.3% (95%CI, 17.2–59.3%) and an AUC of 82% (95%CI, 69–96%), resulting in low accuracy as compared with other methods (CEUS and 3D-US).

58 out of the 71 vulnerable plaques (histological confirmed) were demonstrated at CDUS.

27 out of the 30 plaques were confirmed as stable, with three false positive cases.

These results determined a sensitivity of 81.7% (95%CI, 70.7–89.9%), a specificity of 90.0% (95%CI, 73.5–97.9%), a PPV of 95.1% (95%CI, 86.3–99.0%) and a NPV of 67.5% (95%CI, 50.9–81.4%), with a total AUC of 86% (95%CI, 79–93%).

### 3D arterial analysis software

88 out of the 91 plaques with high grade of stenosis (CTA confirmed) were demonstrated at 3D US, with a sensitivity of 96.7% (95%CI, 90.7–99.3%), a specificity of 100% (95%CI, 69.2–100%), a PPV of 100% (95%CI, 95.9–100%), a NPV of 76.9% (95%CI, 46.2–95.0%) and an AUC of 98% (95%CI, 97–100%; there was no significant difference as compared to the results of CTA.

62 out of the 71 vulnerable plaques (histologically confirmed) were demonstrated at 3D US.

28 out of the 30 plaques were confirmed as stable, with two false positive cases.

These results determined a sensitivity of 87.3% (95%CI, 77.3–94.0%), a specificity of 93.3% (95%CI, 77.9–99.2%), a PPV of 96.9% (95%CI, 89.2–99.6%) and a NPV of 75.7% (95%CI, 58.8–88.2%), with a total AUC of 90% (95%CI, 84–96%).

### CEUS

81 out of the 91 plaques with high grade of stenosis (CTA confirmed) were demonstrated at CEUS, with a sensitivity of 89.0% (95%CI, 80.7–94.6%), a specificity of 100% (95%CI, 69.2–100%), a PPV of 100% (95%CI, 95.5–100%), a NPV of 50.0% (95%CI, 27.2–72.8%) and an AUC of 95% (95%CI, 91–98%), revealing no significant differences relative to CTA;

64 out of the 71 vulnerable plaques (histologically confirmed) were demonstrated at CEUS;

29 out of 30 plaques were confirmed as stable, with only one false positive case.

These results determined a sensitivity of 90.1% (95%CI, 80.7–95.9%), a specificity of 96.7% (95%CI, 82.8–99.9%), a PPV of 98.5% (95%CI, 91.7–100%) and a NPV of 80.6% (95%CI, 64.0–91.8%), with a total AUC of 93% (95%CI, 89–98%).

### Morphological and immunohistochemical evaluation

71/101 (70%) carotid plaques were considered vulnerable at histological examination: 25 plaques (35%) with moderate (16) or extensive (9) lipid core, 23 plaques (32%) with moderate (14) or extensive (9) inflammatory infiltrate mainly consisting of macrophages, 23 plaques (32%) with high value of micro-vessel density, in particular in 9 associated intraplaque hemorrhage.

No side effects due to all the diagnostic methods were registered.

Results are reported in Table 1 (grade of stenosis) and Table 2 (grade of vulnerability).

**Table 1:** Diagnostic performance of each methods for carotid stenosis degree evaluation

	STENOTIC PLAQUES	NONSTENPLAQUES	SENS (95% CI)	SPECIF (95% CI)	PPV (95% CI)	NPV (95% CI)	AUC (95% CI)
CDUS							
Pos	77	2	84.6% (75.5%-91.3%)	80.0% (44.4%-97.5%)	97.5% (91.2%-99.7%)	36.3% (17.2%-59.3%)	82% (69%-96%)
Neg	14	8					
3DUS							
Pos	88	0	96.7% (90.7%-99.3%)	100% (69.2%-100%)	100% (95.9%-100%)	76.9% (46.2%-95.0%)	98% (97%-100%)
Neg	3	10					
CEUS							
Pos	81	0	89.0% (80.7%-94.6%)	100% (69.2%-100%)	100% (95.5%-100%)	50.0% (27.2%-72.8%)	95% (91%-98%)
Neg	10	10

**Table 2:** Diagnostic performance of each method for evaluation of carotid plaque vulnerability

	VULN. PLAQUES	STABLE PLAQUES	SENS (95% CI)	SPECIF (95% CI)	PPV (95% CI)	NPV (95% CI)	AUC (95% CI)
CDUS							
Pos	58	3	81.7% (70.7%-89.9%)	90.0% (73.5%-97.9%)	95.1% (86.3%-99.0%)	67.5% (50.9%-81.4%)	86% (79%-93%)
Neg	13	27					
3DUS							
Pos	62	2	87.3% (77.3%-94.0%)	93.3% (77.9%-99.2%)	96.9% (89.2%-99.6%)	75.7% (58.8%-88.2%)	90% (84%-96%)
Neg	9	28					
CEUS							
Pos	64	1	90.1% (80.7%-95.9%)	96.7% (82.8%-99.9%)	98.5% (91.7%-100%)	80.6% (64.0%-91.8%)	93% (89%-98%)
Neg	7	29					
CTA							
Pos	62	1	87.3% (77.3%-94.0%)	96.7% (82.8%-99.9%)	98.4% (91.5%-100%)	76.3% (59.8%-88.6%)	92% (87%-97%)
Neg	9	29					

**Fig. 5:**
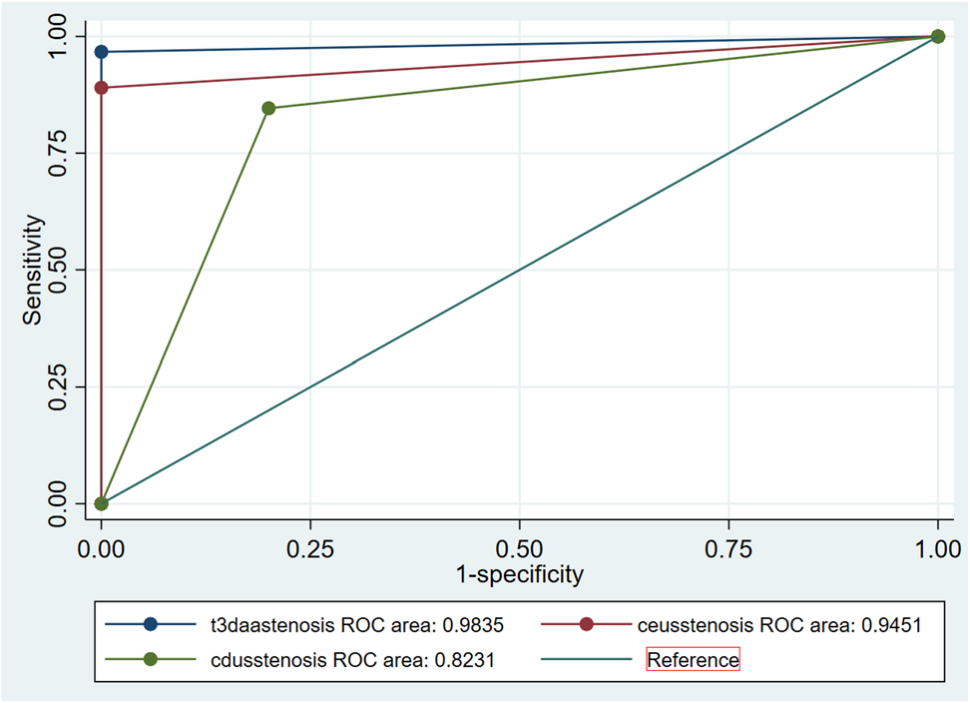
ROC curves comparison for carotid stenosis degree evaluation

The comparison of AUCs for carotid stenosis evaluation demonstrated the accuracy of all ultrasound methods in assessing the carotid stenosis degree, in particular, CEUS and 3DAA (Fig. 5). Of note, 3DAA resulted the most accurate US technique in measuring carotid stenosis and this difference was statistically significant compared to the CDUS (p < 0.05) at Bonferroni test (Table 3).

**Table 3:** Comparison US methods versus CTA (gold-standard) for carotid stenosis degree evaluation by AUROC and Bonferroni test

	ROC Area	Bonferroni Pr > Chi^2^
CDUS (standard)	,8231	
3DAA	,9835	,0396
CEUS	,9451	,1418

**Fig. 6:**
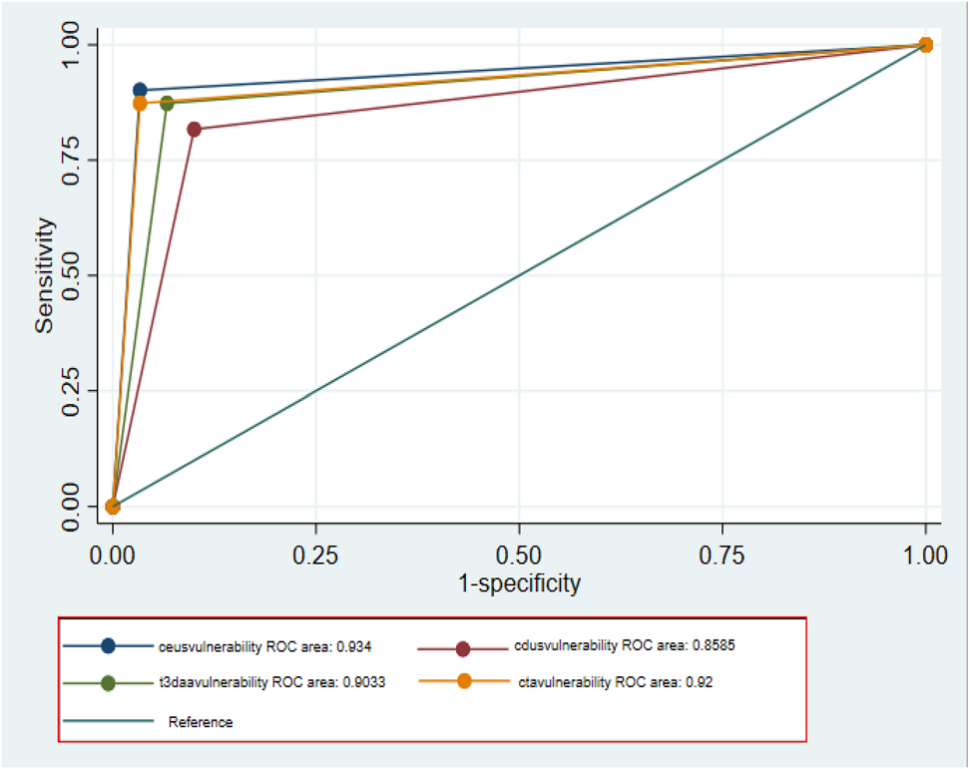
ROC curves comparison for vulnerable carotid plaque evaluation

Similar results were observed regarding plaque vulnerability and there was an excellent concordance between 3DAA, CEUS and CTA (Fig. **6**). CEUS has been shown to be the superior modality for detecting plaque characterization; the difference is statistically significant compared to CDUS (p < 0.05) that is the least accurate (Table 4).

**Table 4:** Comparison imaging methods versus Histology (standard) for evaluation of carotid plaque vulnerability by AUROC and Bonferroni test

	ROC Area	Bonferroni Pr > Chi^2^
CEUS (standard)	,9340	
3DAA	,9033	,3377
CTA	,9200	,4630
CDUS	,8585	,0240

## Discussion

The clinical reliance on the anatomic definition of the degree of carotid artery plaque stenosis has notably limitations, whereas, newer methods incorporating physiologic parameters of the vulnerable plaque enhance plaque characterization and result in improved diagnostic accuracies. Based upon current professional society guidelines, carotid artery revascularization is recommended to treat asymptomatic carotid artery stenosis of 60–99% only in the presence of one or more characteristics that may be associated with an increased risk of late ipsilateral CVA [1, 2]. It is therefore mandatory to identify imaging criteria that might confer an increased risk of CVA on BMT [7].

Over the last few years, the vulnerability features of atherosclerotic plaque have been extensively studied for the estimation of CVA risk especially in asymptomatic individuals that are eligible for medical treatment or revascularization. In fact, the ipsilateral ischemic cerebrovascular events are more common in patients with high-risk plaques (4.3 events per 100 person-years) than in those without high-risk plaques (1.2 events per 100 person-years), with an odds ratio of 3.0 [32, 33].

Ulceration, neovascularization, inflammation, thin fibrous cap, lipid core, intraplaque hemorrhage are all considered causes of plaque vulnerability [34–36].

Most notably, investigators have identified that intraplaque neovascularization (IPN) and hemorrhage are histopathological features associated with a vulnerable plaque that in case of rupture could result in a CVA [37–39].

In the recent Rizvi’s meta-analysis [40], intraplaque hemorrhage (IPH), lipid-rich necrotic core (LRNC) and thin/ruptured fibrous cap (TRFC) found on MR are associated, respectively, with 11.9%, 5.4%, and 5.7% annual rate of ischemic events and increased risk of future ischemic events (OR of 6.37, 4.34, 10.60, respectively).

These histopathological features should be identified with diagnostic methods which are reliable and at the same time less invasive as possible.

CDUS imaging is well-established method used to assess the degree of carotid stenosis and permits delineation the plaque border and assessment of plaque echogenicity. Although plaque hypoechogenicity is a strong marker of vulnerable plaque as the sonographic equivalent of a LRNC and IPH, B-mode characterization of carotid plaques vulnerability is limited by poor inter- and intra-observer agreement, as well as poor signal-to-noise ratio [41].

CEUS is a well-established method to assess vascularization, with several clinical applications (i.e., neoplastic lesion, blunt trauma, inflammation, aortic endoleaks after EVAR [42–49]). Since plaque neovascularization has been a consistent feature of plaque vulnerability, many authors confirmed the correlation between high risk or vulnerable plaques and contrast enhancement with associated intraplaque neovascularization [50, 51] and inflammatory processes. High grade of contrast enhancement appears to be directly related to increased inflammatory infiltrate; similarly, Owen reported that late-phase contrast enhancement of plaque was associated with inflammatory plaque infiltration [26].

Feinstein reported that early-phase CEUS could identify plaque neovascularity and provides an enhanced delineation of plaque anatomy including ulcerations in comparison to gray-scale or Color-Doppler imaging [52].

Similar to prior studies, we observed that the neovascularization detected by intraplaque enhancement on CEUS was strongly associated with the number of newly formed microvessels originating from adventitial vasa vasorum based on histology specimens from post-endarterectomy plaques [53]. Importantly, SonoVue remains a blood pool agent such that microbubbles remain within the vascular space and do not enter the extravascular space [54]. Hoogi [55] demonstrated an indirect correlation between contrast enhancement and the degree of inflammatory infiltrate. Conversely, Li et al. [25] failed to observe a correlation between contrast enhancement and inflammatory infiltration (CD68).

The most recent EFSUMB guideline strongly recommends CEUS use in carotid stenosis to better differentiate between total carotid occlusion from carotid sub occlusion along with identification of intraplaque neovascularization [56].

The 3D arterial analysis applies a directional force to the tissue to cause deformation of the carotid wall. The result is a colorimetric map based on the tissue stiffness, providing a representation of vulnerable areas, and thus delivering a stratified evaluation of the risk. In our experience, 3D arterial analysis provides a reliable morphological reconstruction used to assess plaque shape, and luminal surfaces along the vessel axis, thus allowing an evaluation of the plaque’s volume and degree of stenosis. The main disadvantage of the 3D technique includes the physical size and weight of the probe. It is wider than the 2D array and requires a software to process a 3D image [57].

Unlike previous studies that attempt to assess plaque vulnerability on B-mode images with Gray-Scale Median Analysis [58], our study utilized 3D carotid plaque assessment and included technology to assess tissue stiffness used to identify soft and vulnerable plaques.

To date, a recent published reports used 3DUS for carotid wall assessment and focused on volumetric monitoring of the carotid plaque in relation to ongoing atherosclerosis treatment.

Schreinlechner demonstrated the correlation between the inflammation marker called neutrophil gelatinase-associated lipocalin (NGAL), and the carotid plaque volume when measured using 3DUS in 323 asymptomatic patients [59].

Noflatscher showed the carotid plaque reduction with 3DUS probe in subjects following a diet based on vegetables, juice and fish [60]. Notably, Chen demonstrated a reduced progression of plaque volume in patients taking statin therapy versus a control group (no statin therapy) (+ 70 mm3 vs + 15mm3, P < 0.05) [61]. López-Melgar demonstrated the association of carotid plaque volume using three-dimensional vascular ultrasound with subclinical cardiovascular disease in patients with metabolic syndrome system. In fact, the carotid plaque burden is directly associated with cardiovascular risk (r = 0.308; p = 0.032) [62].

Song demonstrated positive correlation between 3D-US and CTA in the volume measurement of 139 carotid plaques (148.5 ± 133.0 mm3 vs. 154.1 ± 134.6 mm3, R: 0.9825, P = 0.998, P-value for r < 0.001). Furthermore, 3DUS performed better than 2D-US in the plaque detection, finding 108/139 (78%) and 70/139 plaques (50.4%), respectively [63].

Regarding plaque characterization, ulceration is identified as a feature of vulnerable plaques that increases the risk of CVA [64]. Artas demonstrated that 3DUS highlights plaque irregularities and ulcerations and performed outperformed 2DUS (p > 0.05) and included enhanced inter-observer agreement (k coefficients: 0.95) [65]. Similar results were reported by Heliopoulos, with 16.1% plaque ulcerations using 3DUS versus 6.5% with CDUS (P = 0.125); the inter-observer agreement was significant (κ = 0.973, P < 0.001 for 3D, and κ = 0.885, P < 0.001 for 2D) [66].

Several other authors compared 3DUS with other imaging methods, in particular:

1. Kozàkovà reported increased diagnostic accuracy of 3D US as compared to carotid angiography regarding sensitivity, specificity and diagnostic accuracy values of 96.0%, 77.7% and 88.3%, respectively. In particular 46 carotid stenosis were studied by 3D vascular system and biplane carotid angiography with an excellent correlation between the two methods (r = 0.79, P < 0.01) and with higher values associated with stenosis between 40 and 70% [67].
2. Pelz et al., reported a corresponding agreement between 3DUS and MRA in detection of carotid artery stenoses with an er-rater reliability of 0.75 ± 0.23 for the common carotid artery and 0.78 ± 0.21 (n = 92) for the ICA [68]. And Igase et al., observed 13 carotid plaque ulcerations, whereas, MRA imaging identified only 2 out of a total of 33 carotid artery plaques. [69].

As is reported in the last European guidelines [30], MRI is a useful non-invasive imaging technique for the plaque characterization especially identifying the "high-risk" features of the carotid plaque such as the lipid-rich necrotic core or intraplaque hemorrhage [70]. Furthermore, the intravenous administration of Gadolinium (Gd) also allows to evaluate the plaque neovascularization and to differentiate a necrotic core from an surface ulceration [71]. However, considering the high cost and MR contraindications is not still widespread universally used.

Conversely, CDUS is generally the first line imaging modality for carotid stenosis evaluation but its accuracy is limited for plaque vulnerability assessment. So the recent EFSUMB guidelines, to overcome these limitations, strongly recommends CEUS to differentiate between total or near total occlusion of carotid tight stenosis and to evaluate the carotid plaque neovascularization. The limitation of CEUS represented by limited evaluation of plaques content. More recently interesting new US software was introduced such as 3D arterial analysis that automatically measures the carotid stenosis volume and depicts the vulnerable areas according to the plaque composition.

The main advantages of the ultrasound methods include well-established user base and availability, low cost, real-time assessment of disease, established safety profiles and no use of ionizing radiation. And unique among all imaging modalities, CEUS offers unparalleled spatial and temporal resolution of arterial vasa vasorum and intraplaque neovascularization. It is this unique aspect of CEUS that permits direct assessment of plaque neovascularization in "high-risk" patients and is not accessible with other imaging modalities [25, 51] including CTA and MRA and PET [72].

The main disadvantages of the standard ultrasound methods include lack of volumetric analyses, variability due to operator dependence, and the ability to differentiate lipid core from intraplaque hemorrhage.

Consistent with published reports, our experience confirms that different diagnostic techniques have similar accuracies regarding the measurement of the carotid stenosis degree.

Color-Doppler is useful in identifying the carotid stenosis degree both as a direct evaluation of the blood flow and the patent lumen and as a quantification of velocimetric parameters such as PSV and EDV related to hemodynamic significance.

However, Doppler evaluation may underestimate carotid stenoses showing velocimetric values lower than those expected from significant stenoses when compared to other diagnostic methods. This scenario particularly occurs if the plaque extends along the carotid vessel lumen and/or if the plaque is very hypoechoic and vascularized; perhaps due to the plaque composition as compared to a fibro-calcific plaque [53].

3DUS may overcome the known limitations of using two-dimensional imaging based on the use of 3D volume acquisitions avoiding partial volume effects. However, as currently configured, the 3D probes are rather oversized, limiting the ability to evaluate highly hypoechoic plaques.

CTA is the standard for the quantification of carotid stenosis by evaluating not only the carotid vessels but also the aortic arch and cerebral circulation. The major limitation of CTA is the beam-hardening artifact due to vessel wall calcifications, radiation exposure and the use of a potentially nephrotoxic iodinate contrast use.

There is continued interest in utilizing novel markers of plaque vulnerability. Ulceration is defined as an irregularity of the plaque surface with a depression at least 2 mm in depth [21], with a well-defined back wall, and it is clearly visible with ultrasound, CTA and MRA methods [27]. In particular, CEUS results excellent in the carotid ulceration detection [23, 52].

Plaque neovascularization is an important histologically proven vulnerability parameter that can be optimally assessed by CEUS with great accuracy [13, 25, 26].

CTA is less efficient for intraplaque neovascularization, likely due to the study protocols which are based on the arterial phase that is temporally not applicable for late plaque enhancement.

The present study demonstrated that CDUS has lower sensitivity and a specificity (respectively, 84,6% and 80%) than other methods in the evaluation of plaque stenosis, especially in comparison with CTA; alternatively, 3D arterial analysis, and CEUS represent a sensitivity of 96.7% and 89%, respectively, with a specificity of 100%; similar to CTA results, in the evaluation of the stenosis degree. Indeed, according to our results, 3D arterial analysis and CEUS have a higher diagnostic accuracy than reported in literature, when compared with CTA used as the standard.

Regarding plaque vulnerability, CDUS sensitivity and specificity (respectively, 81.7% and 90%) are lower than CEUS, CTA and 3D arterial analysis sensitivity and specificity (varied between 87.3% and 90.1% and 93.3.% and 96.7%, respectively). Results show that CDUS diagnostic accuracy is lower than 3D arterial analysis and CEUS ones; in particular, CEUS shows enhanced efficacy for recognition of vulnerable plaque with results similar to histology.

Combination of 3D arterial analysis and CEUS is a cost-effective modality to assess plaque morphology and characteristic with a positive statistical agreement with CTA findings. Thus, this multiparametric ultrasonography method can be clinically useful in planning the optimal management of the carotid artery disease.

Indeed, multiparametric US study with CEUS and 3D arterial analysis is a viable solution for patients with relative or absolute contraindications to iodinated contrast medium or in potentially high-risk patients with a carotid stenosis of less than 50% of lumen.

The limitations of this study are represented by the following: (a) small population size, (b) single center site, (c) limited patient selection (surgical candidates only), and (d) use of a two-phases CT protocol involving a pre-contrast phase (no use of dual energy CT).

## Conclusion

Multiparametric ultrasonography may be considered a clinical option for the assessment and optimal management of carotid artery disease in patients. Large multi-center prospective studies addressing the multiparametric US findings with patient outcomes in carotid atherosclerotic disease are warranted to establish the diagnostic clinical utility.

## Abbreviations

3DAA: 3D arterial analysis
AUC: Area under the curve
BMT: Best medical therapy
CDUS: Color-Doppler ultrasound
CVA: Cerebrovascular accident
CEUS: Contrasted enhanced ultrasonography
CTA: CT angiography
FC: Thin/ruptured fibrous cap
IPH: Intraplaque hemorrhage
IPN: Intraplaque neovascularization
LRNC: Lipid-rich necrotic core
MPUS: Multiparametric ultrasound
MRA: MR angiography
NPV: Negative predictive value
PPV: Positive predictive value
SS: Steady state
US: Ultrasound
